# Controversy: Is There a Role for Adjuvants in the Management of Male Pattern Hair Loss?

**DOI:** 10.4103/0974-2077.69016

**Published:** 2010

**Authors:** Rajendrasingh J Rajput

**Affiliations:** *R R Institute, 201-A, Gasper Enclave, Ambedkar Road, Pali Hill Market, Bandra (W), Mumbai 400050, India*

**Keywords:** Antioxidants, cyclical medicine, low-level laser

## Abstract

Patients with hair loss are seeking treatment at a younger age and during earlier stages. Not all need hair transplants. Because of the lack of assured management and the fear of side-effects, patients are turning to ineffective alternative remedies from self-claimed experts. In this report, we discuss the available treatment options and how best they can be used in combination to produce satisfactory results. The traditional approach consists of administration of drugs such as minoxidil and finasteride. We propose a hypothesis that nutritional supplements, 2% ketoconazole shampoo and low-level laser therapy along with finasteride 1 mg used once in 3 days with 2% minoxidil used everyday, given in a cyclical medicine program may be useful to manage hair loss and achieve new hair growth. The scientific rationale for such an approach is explained. The need for further studies to establish the efficacy of the regime is stressed upon.

## INTRODUCTION

Androgenetic hair loss often begins as early as 14 years, with 11% of the patients being below the age of 20 years. The incidence of hair loss in the age group of 26–45 years is 40% and rises to 57% at 60 years, although at this age it is perceived as a part of natural ageing. The number of men who seek treatment is five times as compared to women. Psychological and social embarrassment, stress and depression due to hair loss are seen in 46% of the patients.[1–3] Patients often resort to alternative therapies due to the lack of awareness, lack of guaranteed and consistent results with drugs, the need for prolonged or lifelong use of medication and the fear of side-effects.[3] The existing available treatment options often do not meet the expectations of our patients.

Androgenetic alopecia (AGA) is caused by twin factors of heredity and male hormone, showing polymorphism with variable expression, early or late in onset and with slow or rapid progress. The androgen gene confers only 40% of male pattern baldness (MPB). Other genes controlling hair follicle cycle, response to metabolic states, cell division, stress and environmental factors confer the other 60%.[4,5] Because AGA is not entirely genetic, it is referred to as male pattern hair loss (MPHL) and female pattern hair loss (FPHL) instead of AGA. Current treatments for hair loss are based on the beneficial sideeffects of pharmacological agents.[4] Questions have been raised whether factors other than androgens have a role in MPHL. There is some evidence to show that it may be influenced by environmental and lifestyle factors in men and women.[4,5] It is also known that antiandrogen (finasteride)-induced inhibition of 5-alpha reductase (AR) does not help in all cases with AGA, suggesting the possible role of other possible causative factors.

In view of these, this article examines the available evidence for the role of alternative factors such as dietary factors and other adjuvants in the management of MPHL and proposes a scheme for its management. It is however stressed that the proposed scheme is based on experimental evidence and extrapolation of such experimental data and, therefore, further large-scale controlled trials are essential for its uniform acceptance.

## INSIGHTS INTO THE MODE OF ACTION OF ANDROGENS

Testosterone-binding globulin, corticosteroid-binding protein and albumin bind 97% testosterone in circulation, while 3% is free testosterone.[6] Type I 5-AR is available in the pilosebaceous unit.[7] Type II 5-AR is found on the outer root sheath of the hair follicles, prostate and dermal papillae. At all these sites, the testosterone is converted to dihydrotestosterone (DHT),[7] which binds with the cytosol androgen receptor and is carried into the cell nucleus, where it binds to DNA and produces an mRNA leading to miniaturization of the follicles.[6,7] But, there are alternative pathways. Testosterone can be converted to DHT in the circulation and can enter the cell directly without 5-AR or the receptors. Testosterone can find a different cytosol receptor to reach the nucleus and DNA. Other androgens like dehydroepianderosterone sulphate (DHEAS) can also enter the cell directly and get converted to DHT. Diminished levels of enzymes like aromatase, adenylate cyclase and cyclic AMP cause poor hair growth, suggesting the involvement of unknown mechanisms.[6] These may be the reasons why we cannot offer the same benefits to all patients using DHT suppression alone.

## LIMITATIONS OF PHARMACOLOGICAL THERAPY: SIDE-EFFECTS OF MINOXIDIL AND FINASTERIDE

A combination of minoxidil and finasteride is more effective than each of them used alone.[8] However, a minimum period of 4–6 months or more is required before significant clinical improvement is noticed. There are several apprehensions in the minds of patients about the use of drugs. Often, the side-effects of the drugs are exaggerated in the lay media and by practioners of alternative medicine, resulting in poor patient compliance. Several factors therefore need to be considered while starting the treatment:

1. Finasteride 1 mg daily does not show any side-effects in 98% of the cases. Common side-effects of finasteride are reduced libido and reduced seminal volume, seen in 2% of the cases. Of these, 1% is a “nocebo” effect,[9] which improves with continued therapy. This effect is not due to the pharmacological action of the medicine but results from the psychological awareness of the possibility of the side-effects. The other 1% of the side-effects are completely reversible after 5 days of discontinuation of the medicine.[9]
2. The main side-effects of minoxidil include pruritus, allergic dermatitis, palpitation, scalp irritation, worsening of seborrheic dermatitis, and hypertrichosis in women, on the face, arms, chest and sacral area. Minoxidil is preferably avoided in pregnant and breast feeding women.[10,11] Use of new propylene glycol-free, minoxidil gel and foam has reduced these side-effects.[12]

Thus, it can be seen that serious side-effects due to these drugs are rare. However, a large number of patients stop the treatment after a few months. Hence, there is a need for more effective and alternate therapies.

## ALTERNATE HYPOTHESIS FOR ANDROGENETIC ALOPECIA

While the role of androgens is undisputed, other hypotheses have been proposed:

1. Oxidative stress directly affects the cell membrane[13,14] and facilitates entry of DHT, DHEAS and other damaging factors into the cell. Reactive oxygen species (ROS) cause sebaceous gland hyperplasia, promoting increased type I 5-AR enzyme activity and higher DHT formation.[15] Action of androgens is mediated through increased generation of superoxide, which is neutralized by super oxide dismutase (SOD).[15] SOD mimetics are used to reverse miniaturization, e.g. copper binding peptide, prazotide copper.[16] The SOD in the mitochondria contains manganese (Mn SOD). SOD in the cytoplasm contains copper and zinc (CuZnSOD).[15] All these minerals are essential for hair growth. Active small molecule antioxidants are ascorbic acid (vitamin C), tocopherol, lipoic acid, uric acid, glutathione and polyphenol. Antioxidants scavenge-free radicals.[15]
  Lipid-soluble and water-soluble small molecule antioxidants act in the extracellular space.[15,17] These are alpha-tocopherol (vitamin E 100–200 IU per day), beta-carotene, carotenoids, alpha-carotene, lycopene, lutein and zeaxanthine. Vitamin C, the water-soluble antioxidant, helps in biosynthesis[15] and is the first-line antioxidant in plasma against different ROS.[15] Other useful scavengers are pyridine-N-oxides such as 3-carboxylic acid pyridine-N-oxide and its esters.[15] Nitric oxide (NO) initiates and maintains hair growth while superoxide inhibits hair growth. Use of arginine can enhance NO and promote angiogenesis for new hair growth.[15]
  All these evidence show that there may be a role for oxidative stress in hair loss.
2. Another study suggests an alternate mechanism of action of DHT resulting in miniaturization of hair follicles. According to this hypothesis, the adult cranial bones, especially the frontal and parietal bones, continue to grow in size even in adulthood under the influence of DHT, thus resulting in bone expansion and remodeling, which, in turn, compromises the blood flow through the capillary network in these areas and thus initiates miniaturization of hair follicles in the affected area.[18]
3. There is evidence suggesting that regular aerobics and weight training can reduce the free testosterone level in the blood in such people and thus have a beneficial effect in pattern hair loss.[19,20] But, only weight training without aerobics can often increase serum free testosterone levels and susceptibility to hair loss.[21–24]

These hypotheses suggest that alternative medical treatments deserve consideration in the management of pattern hair loss. These options are considered below:

### Importance of nutrition for the anagen phase

To re-enter anagen from a resting phase, the dermal papilla cells show a high spurt in cell division and increased growth rate at the onset of the cycle. This requires a good supply of nutrients and a toxin-free environment for the growing cells. If these requirements are not met, the resting phase is prolonged and the growth phase fails to commence.[25] Hair loss is associated with anemia, which may not be detected.[26,27] Low iron levels are compensated by re-absorption of iron from the spleen, bone marrow, etc. In such compensated states, low serum level may not always be identified, but iron is not available for the growing hair.[26,27] Serum ferritin is raised in inflammatory disorders and the ironbinding capacity can be high in a low-iron state due to low percentage saturation.[27] Blood calcium levels are continuously maintained in exchange with bone and hair.[27] Hypoproteinemia is associated with thin, dry, brittle hair and hair loss.[27,28] Antioxidants and nutritional supplements have to be used even in clinically normal levels of iron, calcium, amino acids, vitamins and minerals in order to achieve hair growth. These can be better used in a preventive low dose, once in 3 days, instead of a higher everyday use in therapeutic dosage.

### Possible role of low-level laser therapy

The Hungarian researcher, Mester, in 1967, found that 500 milliwatts, low-power, 694 nm ruby laser increased hair growth on the backs of shaved mice.[29] Possible role of low-level laser therapy (LLLT) has been used to reduce inflammation and enhance wound healing.[29] LLLT is proposed to act by stimulation of mitochondria to produce more ATP and cyclic AMP, with activation of response to oxidative stress,[30–32] displacing NO from the cells and allowing more oxygen to enter. Released NO also induces vasodilatation and improves blood flow to the hair roots. In routine practice, LLLT may not be advised alone but has benefits when used as adjunct to the medicines and also helps in post-transplant cases.[33,34] Another study that used the Hairmax laser comb in patients with AGA also suggested that there was both subjective and objective improvement in the hair density, texture and hair fall.[35]

### Possible role of anti-inflammatory medications in the treatment of MPHL

In AGA, scalp biopsies show decreased anagen hair and increased vellus hair. There is perifollicular lymphocytic infiltration and perifollicular concentric fibrosis around the upper and lower follicles,[36] suggesting that antiinflammatory treatments may benefit such patients.

### Role of ketoconazole

2% ketoconazole shampoo is an antifungal effective in seborrheic dermatitis, has an anti-inflammatory effect and reduces Malassezia colonization of the scalp. It also has local DHT suppression activity, which may be useful in the management of pattern hair loss.[37,38]

## HYPOTHESIS FOR AN ALTERNATIVE REGIMEN IN PATTERN HAIR LOSS

Based on these hypotheses, we propose an alternative regime as follows. The principles of this proposed regime are:

- To prevent the hair from miniaturization using finasteride, promote hair growth using minoxidil and support growing cells using antioxidants, vitamins, iron, folic acid, biotin, calcium, minerals and amino acids.
- Another principle is to use the least-effective doses of drugs to minimize their side-effects, which helps to allay the anxiety of patients about sideeffects and leads to better compliance. Recalling original finasteride dose studies, even 0.2 mg per day caused 55% DHT suppression, while 5 mg per day achieved 69% DHT suppression.[39] The plasma half-life of finasteride is 6–8 h and tissue binding is 4–5 days. Considering these facts, we propose that finasteride 1mg be used once in 3 days. This approach gains confidence of the patients that the low dose and the break of 2 days will keep them free of side-effects. Minoxidil, ketoconazole shampoo, antioxidants and nutritional supplements are combined with this twice-weekly finasteride.

## DIETARY AND LIFE STYLE MODIFICATIONS TO IMPROVE HAIR GROWTH

Masumi Inaba of Japan has shown a cause and effect relationship between the diet and the severity of hair loss through documentation and scalp biopsies.[27] Fried foods and red meat are avoided to reduce the overall activity of oil and sebum glands, as these are the sites for 5-AR enzyme activity, and hyperactive glands may lead to more conversion of DHT.[13] It also reduces the accumulation of free radicals, which are harmful to rapidly dividing cells. Next is to avoid sugar-based foods, such as chocolates, pastries and sweets; however, some sugar in tea or coffee is permitted.[27,28] Increased sugar leads to insulin release, which, in turn, causes release of testosterone from its binding protein and makes it available for conversion to DHT. High-fat foods, fried foods and bakery products are avoided. Foods with artificial flavours, taste makers, additives, preservatives and colas are avoided as these chemicals lead to the formation of free radicals in the body.[27] Chinese foods made with aginomoto may adversely affect the hair.[27] Sprouts, green leafy vegetables, pulses and nuts along with plenty of water a day make up for balanced diet and provide all the nutrients required for healthy and glowing hair.

Avoidance of smoking can be beneficial in hair loss, as nicotine is known to decrease blood flow to the hair follicles by causing vasoconstriction and also leads to accumulation of free radicals in the hair roots thus damaging hair roots.[27] As stated earlier, aerobic exercises may help reduce serum androgen levels[19,20] and, therefore, are advocated.

## RESULTS OF OUR EXPERIENCE WITH CYCLICAL THERAPY

A randomized control trial was conducted with four groups of men and women in all ages and all grades of hair loss[40]. The study included 500 patients randomly selected, irrespective of age, sex and grade of hair loss. The progress was recorded by digital photographs, folliscopic computerized analysis for density counts per square centimeter and measurement of hair caliber in microns. Patients also had a self-assessment score. The trial showed that the cyclical medicine program was effective. Patients had visible improvement in 2 months and good results in 4 months. Improvement continued as the therapy was continued further. New hair growth was recorded till 18 months. Improvement in hair count at 4 months was 30–52%. Improvement in hair caliber at 4 months was 37–47%. Vellus hair count, which initially was 12–50%, decreased to 5–20% at 4 months.

## SUMMARY

Pattern hair loss, although a global problem, has very limited options available for satisfactory treatment. Conventional minoxidil and finasteride are the only two scientifically proven drugs available, but both need long-term compliance from the patients’ side. We seek to hypothesise that antioxidants, diet, exercise and lowlevel laser can be used as adjuvants in combination with minimal doses of finasteride for better compliance and greater efficacy. We also suggest that controlled trials need to be conducted to further establish this hypothesis.
